# Isoconversional Kinetic Analysis and ANN-Based Prediction of Metformin Pyrolysis for Sustainable Waste Management

**DOI:** 10.1021/acsomega.5c03868

**Published:** 2025-10-07

**Authors:** Ramesh Potnuri, Maheswata Lenka, Chinta Sankar Rao, Harshini Dasari

**Affiliations:** † Control Systems & Machine Learning Research Laboratory, Department of Chemical Engineering, 119680 National Institute of Technology Karnataka, Surathkal, Mangalore, Karnataka 575025, India; ‡ Chemical Engineering Department, 125853 Manipal Institute of Technology, Manipal Academy of Higher Education, Manipal, Udupi, Karnataka 576104, India

## Abstract

Pharmaceutical waste poses a growing environmental concern due to its persistence and potential ecological impacts, necessitating effective and sustainable management strategies. This study investigates the pyrolysis of metformin as a means to valorize pharmaceutical waste within a circular economy framework. Pyrolysis experiments conducted on 500 mg of metformin demonstrated the formation of liquid-phase products, characterized by GC–MS, which revealed a high concentration of the active pharmaceutical ingredient (API) alongside carbonaceous, nitro, and acidic compounds. Comprehensive thermogravimetric analyses at heating rates of 10, 20, 30, and 40 °C/min were performed to evaluate the thermal decomposition behavior. Kinetic parameters were determined using four isoconversional methods, namely KAS, FWO, Starink, and FRD, yielding average activation energies of 101.4, 105.8, 101.4, and 111.1 kJ/mol, respectively. Thermodynamic parameters (Δ*G*, Δ*H*, and Δ*S*) were also calculated to gain further insights into the decomposition process. Additionally, an ANN model was developed using temperature and heating rate as inputs to predict mass loss, achieving accurate estimations with an optimized architecture comprising two hidden layers. GC–MS analysis of the pyrolysis liquid identified a high concentration of the API, along with carbonaceous, nitro, and acidic compounds. These findings highlight the potential for API recovery and reuse, as well as the valorization of byproducts for energy or chemical synthesis. The potential recovery of APIs for reuse and the utilization of byproducts as fuels or chemical precursors underscore pyrolysis as a promising route for sustainable pharmaceutical waste management and circular economy integration.

## Graphical abstract

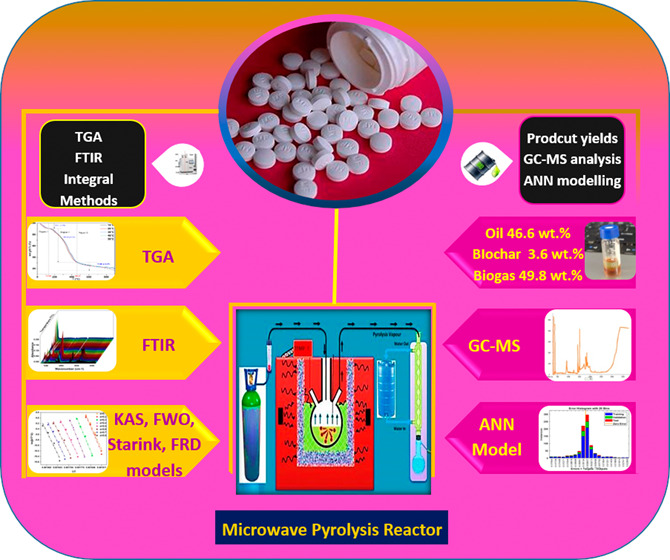

## 1 Introduction

The consumption of pharmaceuticals is growing worldwide, with total global revenues increasing from 0.887 trillion USD in 2010 to 1.42 trillion USD in 2021. The total value will reach around 1.79 trillion USD in 2026.^1^ Similar trends are expected to develop in India since it is estimated that sales of prescription pharmaceuticals will rise from about USD 0.125 trillion in 2016 to over USD 0.273 trillion in 2024.^1^ However, not every pharmaceutical purchased is utilized because of a variety of factors, including poor patient compliance, adverse drug reactions, or the death of a patient.^2^ Due to this, significant amounts of expired, unused, and unwanted prescriptions now exist. In the United States alone, up to one billion USD worth of drugs is thrown out each year as regular household waste.^3^ The aforementioned problems have become more challenging due to the COVID-19 pandemic.^4^ Panic buying resulted from a rise in purchasing multiple categories of pharmaceuticals globally.^3,5^

This has raised issues related to producing even more pharmaceutical waste. Most frequently, these wastes are dumped with municipal solid waste or flushed down the bathroom sink and then enter garbage dumps and the drainage system.^6,7^ They gradually enter the ground via landfill leachate and the environment because wastewater treatment facilities lack the proper equipment to eliminate pharmaceuticals.^8^ Additionally, antibiotic resistance, leading to 0.7 million deaths worldwide each year, can be caused by pharmaceutical waste pollution.^9^ Over 600 pharmaceutically active chemicals have reportedly been detected in the environment, according to studies published in the last 20 years on detecting pharmaceutical waste in the environment.^10^

Many initiatives have been attempted to avoid the incorrect disposal of unused and expired pharmaceuticals, including pharmaceutical take-back programs encouraging people to give back unused medicines to healthcare facilities instead of allowing them to expire in their residences.^11,12^ One example, the ″National Pharmaceutical Recycling Association,″ a program that has been operating in France since 1993 and is currently supported by 90% of the country’s pharmacies, reported in 2016 that 11,000 tons of unused pharmaceuticals had been acquired through the program, which is now regularly used by up to 60% of the citizens of the nation.^3^ The U.S. Environmental Protection Agency (EPA) recommends that the general public utilize pharmaceutical take-back schemes that handle prescription drugs as well as over-the-counter drugs, as they provide a secure, practical, and ecologically responsible option to get rid of unused pharmaceuticals.^13^

The most common method to dispose of collected pharmaceuticals as of right now is incineration. The APIs that are present in the waste are effectively destroyed by it.^3^ However, it consumes much energy, demands temperatures over 1200 °C, and emits hazardous substances like SO_*x*_, NO_*x*_, CH_4_, and CO_2_.^14^ On the other hand, recent research looks into recycling pharmaceuticals by extracting the active pharmaceutical ingredient (API). Ibuprofen was recovered from used Brufen pills in a study by E Silva et al. utilizing a solid–liquid extraction technique and solvent used as an ionic liquid. The study shows a 97.9% extraction efficiency using ibuprofen that is pure enough to be reused in subsequent batches of medicines.^15^ The harmful effects, expenses for recycling, and volatility of the utilized solvents present a challenge to the solid–liquid extraction approach employed to recover and recycle APIs from unused pharmaceuticals. Furthermore, recycling the API from “expired” pharmaceuticals may be possible because multiple have been maintained exceptionally well for a very long time after their labeled expiration deadlines.^3^ Examples include acetaminophen, which has been allowed for usage for a maximum of two years after the labeled expiration date.^16^ Even 35 years after the indicated deadline for expiration, theophylline exhibited about 90% stability.^17^ Therefore, it is conceivable that recycling pure APIs, even those from out-of-date pharmaceuticals, and employing them in producing new drugs could prove favorable from an environmental point of view and reduce manufacturing expenses, potentially having a high impact.

A recent study investigated the pyrolysis of paracetamol tablets as an innovative approach for pharmaceutical waste disposal and material recovery within a circular economy framework.^48^ This work utilized thermogravimetric analysis and GC–MS to evaluate the thermal decomposition behavior and to identify recoverable products, including the active pharmaceutical ingredient (API). However, the study was limited to a single pharmaceutical compound and employed only two kinetic models (KAS and FWO). In contrast to this earlier work, the present study investigates the pyrolysis of metformin as a representative pharmaceutical waste and broadens the analytical scope by employing multiple kinetic models, comprehensive thermodynamic analyses, and ANN-based predictive modeling. This integrated methodology offers deeper insights into the thermal decomposition behavior and valorization potential of pharmaceutical waste within a circular economy framework.

The primary objective of this study is to demonstrate that pyrolysis is an effective replacement for burning or discarding medications to recover the API and any additional value-added components. The pyrolysis process is a thermochemical conversion method that involves heating organic materials without oxygen.^18,19^ This process results in the degradation of the feedstock materials and the formation of different products, including solid yield, bio-oil, and syngas.^20,21^ Various pyrolysis methods include fast, flash, slow, solar, and microwave-assisted. A limited number of investigations have examined the utilization of pyrolysis to deal with pharmaceutical waste. A few authors investigated the use of pyrolysis to produce alternative energy sources through the mixture of expired pharmaceuticals with their wrapping materials. They observed that the pyrolysis liquid yield produced by the method has energy characteristics that are close to those of oil yield produced from biomass and has the potential to be utilized as a combustible fuel.^22^ Pyrolysis with GC/MS was effectively employed to identify various volatile thermal decomposition products for different pharmaceutical residues observed as food pollutants.^23^ After carefully examining the available literature, the pharmaceutical wastes for this investigation were metformin pharmaceuticals.

Metformin tablets were used as the feedstock for this study. Since Metformin is the most widely used pharmaceutical for diabetes, available over the counter, it was chosen as the preferred medication. Metformin has replaced other pharmaceuticals for diabetes as the primary option due to its outstanding effectiveness and low cost in controlling diabetes, affecting 90% of the 537 million adults worldwide.^13^ Additionally, compared to most pharmaceuticals, Metformin is not designed to be metabolized by the human body; instead, most of the consumed dose is eliminated via feces and urine.^24^ As a result of its extensive use, Metformin has been regularly found in aquatic systems worldwide since 1999. Metformin is a major contaminant of growing concern due to its documented adverse impacts on aquatic life.^25,26^ Identifying and calculating the thermodynamic and kinetic parameters for the pyrolysis of biomass, plastics, and sludge from sewage waste is crucial for designing, developing, and operating the pyrolysis system.^27,28^ Model-free integral methods have been established to analyze biomass, plastics, and sludge waste pyrolysis kinetics using thermogravimetric analysis (TGA) data.^29,30^

There is limited information on the thermal decomposition and safe treatment of pharmaceutical residues such as Metformin, despite their growing environmental concern. Notably, no kinetic investigations on the pyrolysis of Metformin pharmaceutical waste have been reported so far. This study addresses this critical gap by comprehensively examining the pyrolysis behavior of Metformin. It determines the kinetic parameters (reaction model, pre-exponential factor, and activation energy) and thermodynamic properties (Gibbs free energy, enthalpy change, and entropy change) through TGA using model-free integral methods (KAS, FWO, Starink, and FRD). An artificial neural network (ANN) model was also developed to predict mass loss from TGA data, providing a modern complement to conventional kinetic approaches. Additionally, the characterization of pyrolysis products, particularly bio-oil, using GC–MS and FTIR techniques, offers insights into potential value-added applications. This integrated approach delivers a new understanding of the thermal stability and decomposition pathways of Metformin, supporting the development of safer disposal strategies and valorization routes for pharmaceutical waste. The aim of this study is to investigate the thermal decomposition behavior of Metformin hydrochloride using pyrolysis as a sustainable waste treatment approach, and to explore the potential for resource recovery. The specific objectives are (i) To perform thermogravimetric analysis (TGA) at multiple heating rates to evaluate the thermal decomposition behavior of Metformin hydrochloride (ii) To determine kinetic and thermodynamic parameters using model-free isoconversional methods (iii) To develop a machine learning-based artificial neural network (ANN) model for predicting weight loss during pyrolysis (v) To analyze and characterize the liquid pyrolysis products using GC–MS and FTIR to assess their reuse potential.

## 2 Experimental Methods and Materials

### 2.1 Feedstock Materials

Immediate-release metformin tablets were procured from the health care center at the National Institute of Technology Karnataka (NITK), Surathkal, located in Karnataka, India. The weight of each metformin tablet was 500 mg. Each metformin tablet contains inactive ingredients: microcrystalline cellulose, hydroxypropyl cellulose, magnesium stearate, and sodium starch glycolate.^31^ Utilizing a reliable grinding apparatus, the metformin tablets were meticulously processed into a powder form before experimental runs and analytical procedures, with a range of 10 to 100 μm for the average particle size. The temperature required for pyrolysis was attained using a graphite susceptor powder with a 150 μm particle size. The reactor was a 500 mL round-bottomed flask that contained the feedstock. The spaces between components in the thermocouple, condensation, and oil collection beakers were also sealed with Teflon tape. Gas Chromatography (GC)-grade methanol from a central research facility (NITK, India) was utilized to dilute the oil yield from microwave-assisted pyrolysis for GC–MS analysis.

### 2.2 Thermal Analysis of Feedstock

Thermogravimetric analysis (TGA) is a technique for analyzing how a material responds thermally when exposed to increasing temperatures. The thermal degradation, volatile matter, moisture, fixed carbon, and other thermal properties of a material can all be obtained through TGA. The thermal behavior of metformin pharmaceutical powder samples was examined using the Seiko Exstar TG-DTA 6300 thermogravimetric analyzer. The TGA studies were conducted in an N_2_ atmosphere to keep the testing environment inert. The different heating rates, such as 10 °C/min, 20 °C/min, 30 °C/min, and 40 °C/min, are used with around 10 mg of samples. The weight loss was noticed in temperatures ranging from 30 to 550 °C.

### 2.3 Fourier Transform Infrared Spectroscopy

FTIR spectroscopy (Spectrum 2 FT-IR Spectrometer from PerkinElmer in Singapore) was used to evaluate the chemical and structural properties of the materials. A resolution of 0.5 cm^–1^ was used to capture the spectra. Furthermore, the range of spectra is between 450 and 4000 cm^–1^. Nitrogen (N_2_) was used as the purge gas to remove atmospheric carbon dioxide and water vapors from the sample. Even though each sample was evaluated at three separate sampling places, the test results were essentially identical everywhere.

### 2.4 Pyrolysis Setup and Experimental Procedure

The pyrolysis experiments were conducted in a Samsung microwave oven laboratory standard with a circular aperture on the top portion of the microwave oven. A 23 L modified microwave oven that can produce microwave power in the 100–800 W range. Throughout the pyrolysis method, the actual temperature of the bed of the feedstock mixture was monitored using a microwave-compatible K-type thermocouple. The circular bottom flask was covered in the oven’s low thermal conductivity ceramic wool insulation to maintain a steady temperature during the pyrolysis process. The condensation connections, which were connected to the flask, were used to condense the pyrolysis vapors. Furthermore, N_2_ gas purification was offered to remove any oxygen that might have been present in the feedstock. Figure 1 depicts the detailed framework of the experimental setup in great detail. Our earlier articles contain further details on the experiment’s setup and temperature monitoring.^32^

**Figure 1:**
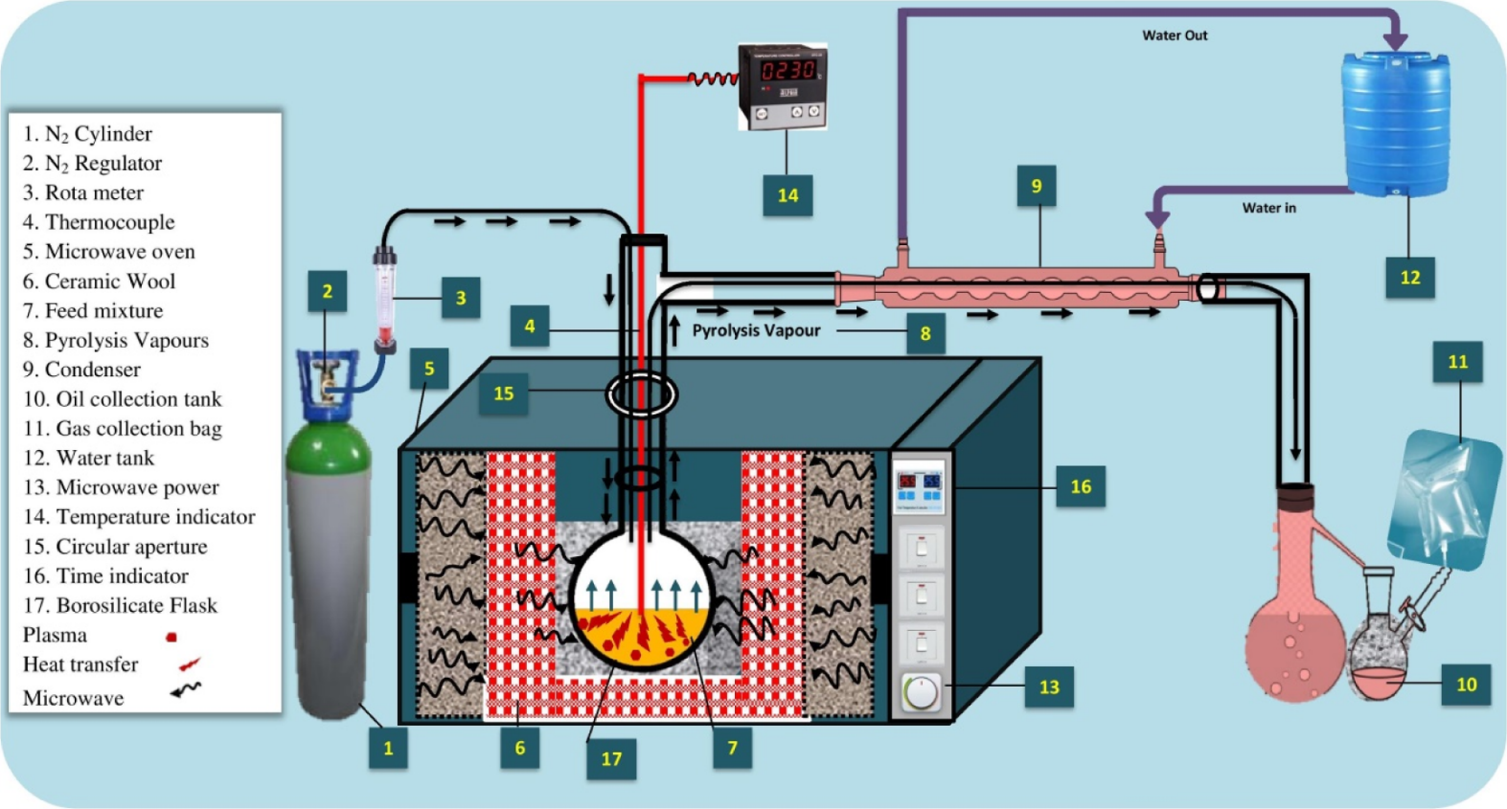
Pyrolysis schematic for metformin decomposition.

A constant microwave power output of 300 W was employed throughout every experiment. Microwave-assisted pyrolysis was performed using a combination of 10 g of susceptor and 10 g of metformin powder to assess pyrolysis product yields. The experiment was conducted three times to ensure accuracy. Microwave pyrolysis reaches a maximum temperature of 550 °C. A 10 min cooling period allowed the pyrolysis reactor to cool to room temperature. An analytical weight balance was used to calculate how much char yield was retained in the reactor for every experiment. High temperatures will not cause a graphite susceptor to melt. Calculating the char yield from the microwave-assisted pyrolysis of the metformin powder is error-free. Following condensation, the quantity of bio-oil yield was collected and quantified. The outlet of the condenser was also attached to a Tedlar bag, which was used to capture the gas yield.

### 2.5 Three-phase Product Yield Calculations

The microwave-assisted pyrolysis experimental setup consists of several parts. The main parts are flasks, condensers, elbow joints, and multiple adapter joints. These parts were weighed before and after every pyrolysis process to calculate the three-phase pyrolysis product yields.^33^

A condenser, elbow joint, multiple adopter joint, and flat-bottom flask variations in weight (*W*) before and after the experiment were included in determining the weight of liquid yield, as illustrated below eq 1.

1 $$W_{liquid}=\left(W_{condenser,after}-W_{condenser,before}\right)+\left(W_{elbowjoint,after}-W_{elbowjoint,before}\right)+\left(W_{adopterjoint,after}-W_{adopterjoint,before}\right)+\left(W_{flask,after}-W_{flask,before}\right)$$

The difference in weight between the round-bottom flasks that were unfilled and filled before and after the experiment was subtracted to determine the weight (*W*) of char yield, as illustrated below eq 2.

2 $$W_{char}=\left(W_{roundbottomflask,after}-W_{roundbottomflask,before}\right)$$

the weight (*W*) of the char and liquid yields was subtracted from the weight of the metformin powder delivered to the reactor to get the gas yield, as shown below in eq 3.

3 $$W_{gas}=\left(W_{Metforminpowder}\right)-\left(W_{liquid}+W_{char}\right)$$

The char yield, bio-oil yield, and gas yields can each be calculated using eq 4, ([Disp-formula eq5]), and ([Disp-formula eq6]), respectively.^34^

4 $$Solid\;yield\left(wt.\%\right)=\frac{Weight\;of\;the\;char\;residue\left(g\right)}{Weight\;of\;metformin\;powder\left(g\right)}\times 100$$

5 $$Liquid\;yield\left(wt.\%\right)=\frac{Weight\;of\;the\;liquid\left(g\right)}{Weight\;of\;metformin\;powder\left(g\right)}\times 100$$

6 Gasyield(wt.%)=100−(Liquidyield(wt.%)+Charyield(wt.%))

### 2.6 Liquid Yield Analysis by GC–MS

Gas Chromatography–Mass Spectrometry (GC–MS) is a highly potent analytical method used to differentiate between volatile and semivolatile substances in chemical compounds without disintegration and determine the ions’ mass and amount. GC–MS is a technique used to analyze pharmaceutical drugs, biofuels, aromatic compounds, natural products, polymers, and more. The LECO Pegasus BT 4D delivers a GC column oven having a 40 °C/min average heating rate and an operating temperature spectrum of 5 to 400 °C. The MS has a detection mass range of 30 to 1500 and a spectrum acquisition rate of 500 spectra per second. The Central Research Facility (CRF), part of the National Institute of Technology Karnataka (NITK) in India, provided all the technical parameters for the GC–MS.

The pyrolysis liquid yields from the two neck flasks were collected after microwave-assisted pyrolysis experiments and blended with 4 mL of gas chromatography-grade methanol. Before analysis, the generated solution was filtered with a 0.2 μm filter to remove any fine particles or other impurities that may damage the GC apparatus. The pyrolysis liquid was analyzed by a split-less sample injector with a capacity of 1 μL, helium as the sweeping gas, and an injector with a temperature of 250 °C. To identify the compounds in the samples, the *m*/*z* peaks corresponding to each peak on the chromatogram were compared to the preidentified compounds stored in the NIST libraries integrated into the MS software. The GC software also generates a mass-match percentage, which shows how well a certain peak on the chromatogram’s mass spectrum matches the spectrum of the substances contained in the software. Furthermore, only peaks with an identical rate of the discovered compound greater than 90% were examined.

## 3 Determination of Kinetic Parameters

During pyrolysis, the main components of the feedstock degrade and blend to generate products with added value. It is challenging to analyze each chemical reaction separately since numerous chemical reactions take place simultaneously and sequentially during the process. Therefore, the following global reaction illustrates the thermal breakdown of the feedstock component.^35^ The reaction can be expressed as follows

7 $${aA}_{\left(s\right)}\to bB_{\left(s\right)}+cC_{\left(g\right)}$$

where *A* is the feedstock component, *B* is char, and *C* is volatile and gas.

When determining kinetic factors from TGA generated under nonisothermal conditions, numerous investigations have employed global reactions that used the model-free kinetic approach. They all described feedstock conversion rates as functions of temperature. The general rate of reaction for the single-step kinetic equation is as follows.^36^

8 $$\frac{d\alpha }{dt}=k\left(T\right)f\left(\alpha \right)$$

where *t* is time, *T* is temperature, *k*(*T*) is the reaction rate constant, α is fractional conversion, and *f*(α) corresponds to the differential mechanism function, which is governed by the underlying reaction mechanism.

The reaction rate constant can be defined using the Arrhenius kinetic expression.

9 $$k\left(T\right)=A\exp\left(\frac{{-E}_{a}}{RT}\right)$$

where, *R* is the universal gas constant, *A* is the pre-exponential factor, *E*_a_ is the activation energy of the reaction at temperature *T*.

The fractional conversion of the feedstock component can be determined by

10 $$\alpha =\left(\frac{w_{0}-w_{t}}{w_{0}-w_{f}}\right)$$

where, *w*_*t*_, *w*_0,_ and *w*_f_ are the sample mass at time *t* the initial sample mass and the final sample mass, respectively.

By substituting eq 9 in [Disp-formula eq8]

11 $$\frac{d\alpha }{dt}=A\exp\left(\frac{-E_{a}}{RT}\right)f\left(\alpha \right)$$

From eq 11, the heating rate can be defined as follows.

12 $$\frac{d\alpha }{dT}=\frac{a}{\beta }f\left(\alpha \right)\exp\left(\frac{-E_{a}}{RT}\right)$$

by integrating eq 12

13 $$g\left(\alpha \right)=\frac{A}{\beta }\int_{0}^{\alpha }\frac{d\alpha }{f\left(\alpha \right)}=\frac{A}{\beta }\int_{T_{0}}^{T}\exp\left(\frac{-E_{a}}{RT}\right)dT$$

Integration in eq 13 does not have an analytical solution. Mainly is solved by the numerical approximation method.^37^ A different model was proposed by different Integral and differential approaches.

### 3.1 Model-Free Integral Methods

Kinetic parameters were determined using isoconversional techniques. According to the main principle, the reaction rate for the thermal degradation of feedstock components depends on the temperature at the specific conversion. A group of TGA kinetics data at various heating rates for the pyrolysis of metformin pharmaceutical powder samples has been utilized to evaluate the activation energy using various isoconversional methods, namely KAS, FRD, FWO and Starink.^38–42^

#### 3.1.1 KAS Method

Through a nonisothermal linear integral, the KAS technique provides a link between heating rate and activation energy. Equation as follows

14 $$\ln\left(\frac{\beta }{T^{2}}\right)=\ln\left[\frac{AE_{a}}{Rg\left(\alpha \right)}\right]-\frac{E_{a}}{RT}$$

The slope of the plot of $\ln\left(\frac{\beta }{T^{2}}\right)$ versus $\frac{1}{T}$ gives a value that can be used to calculate the activation energy. The values of *E*_a_ will vary for a conversion range of 0 to 1. For the overall model average or mean value of *E*_a_ can be determined.

#### 3.1.2 FWO Method

The OFW method is another isoconversional approach that is frequently used in kinetic research. Doyle’s approximation is used by the FWO technique to calculate the activation energy of the pyrolysis process. Equation as follows

15 $$\ln\beta =\ln\left[\frac{AE_{a}}{Rg\left(\alpha \right)}\right]-5.331-1.052\left(\frac{-E_{a}}{RT}\right)$$

slope of ln β vs $\frac{1}{T}$ the plot gives a value of *E*_a_. Values of *E*_a_ will vary for a conversion range of 0 to 1. For the overall model average or mean value of Ea can be determined.

#### 3.1.3 Starink Method

The activation energy for the Starink process can be determined using the following equation.

16 $$\ln\left(\frac{\beta }{T^{1.92}}\right)=Const.-1.0037\frac{E_{a}}{RT}$$

slope of $\ln\left(\frac{\beta }{T^{1.92}}\right)$ vs $\frac{1}{T}$ the plot gives the value of *E*_a_. Values of *E*_a_ will vary for a conversion range of 0 to 1. The overall model average or mean value of *E*_a_ can be determined.

#### 3.1.4 FRD Method

In this method, a 2-step liner fitting process was taken to derive an equation to calculate activation energy. The equation is as follows

17 $$\ln\left(\beta_{i}\frac{d\alpha }{dT}\right)=Const.-\frac{E_{a}}{RT}$$

slope of $\ln\left(\beta_{i}\frac{d\alpha }{dT}\right)$ vs $\frac{1}{T}$ the plot gives a value of *E*_a_. Values of *E*_a_ will vary for the conversion range of 0 to 1. The overall model average or mean value of *E*_a_ can be determined.

#### 3.1.5 Kissinger Method

The frequency factor (*A*) can be determined using the Kissinger approach, which employs the following relation.^1^

18 $$\ln\left(\frac{\beta }{T_{p}^{2}}\right)=-\frac{E_{\alpha }}{RT_{p}}+\ln\left(\frac{AR}{E_{\alpha }}\right)$$

This approach allows for the estimation of *A* by utilizing the activation energy values computed at different conversions from other model-free methods, through eq 19 given by

19 $$A=\frac{\beta E_{\alpha }\exp\left(\frac{E_{\alpha }}{RT_{p}}\right)}{RT_{p}^{2}}$$

where *T*_p_ represents the peak temperature associated with each heating rate.

### 3.2 Determination of Thermodynamic Parameters

The exploration of method viability and estimation of energy for performing the pyrolysis process is assisted by the calculations of thermodynamic parameters.^43^ Δ*H* exposes the energy difference between the raw material and the activated complex by indicating how much heat can be generated or absorbed during a process. Gibbs free energy (Δ*G*) describes the process’s spontaneity. An extensive property that is used to describe a system’s disorder is a change in entropy (Δ*S*).^41^ Using eqs 20, 21, and 22, the thermodynamic parameters Δ*G*, Δ*H*, and Δ*S* are calculated.

20 $$\Delta G=E_{a}+RT\ln\left(\frac{K_{b}T_{p}}{hA}\right)$$

21 $$H=E_{a}-RT$$

22 $$S=\frac{\Delta H-\Delta G}{T_{m}}$$

in eq 18, *K*_b_ represents the Boltzmann constant, h represents the Planck constant, *E*_a_ represent the activation energy, and *T*_p_ is the peak temperature determined by examining a DTG analysis. The activation energy was obtained from integral methods for the estimation of Δ*G*, Δ*H*, and Δ*S* at a certain conversion for five different heating rates (°C/min).

## 4 Results and Discussion

### 4.1 TGA and DTG Analysis of Metformin

Nonisothermal TGA was used to study the thermal degradation of metformin pharmaceutical powder samples. Figure 2a depicts the TGA profile for the thermal breakdown of metformin samples. All heating rates resulted in a similar TGA curve for the metformin samples. At higher heating rates, the main decomposition zone of the curves shifts to higher temperatures, particularly noticeable in the conversion range of approximately 10–80%, attributable to the reduced time available for heat transfer, which affects the onset and peak decomposition temperatures. This is a common TGA phenomenon attributed to the temperature gradient between the surface and the center of the sample particles.^44^ Since the curves are homogeneous in shape and the heating rate does not affect the thermal degradation, the lower heating rates can be employed to maximize the pyrolysis of the pharmaceutical substance. The pharmaceutical powder sample of Metformin exhibited a dominant single decomposition stage on the TGA curve. While minor features were observed in the DTG curve, the main peak was considered representative of a single major reaction for kinetic analysis. The major breakdown started around 217.4 °C and extended slightly beyond 357.3 °C when the heating rate of 10 °C/min was considered. Following this stage was a normal tail, which showed slight mass loss up to a temperature of 507 °C and produced a residual mass.

**Figure 2:**
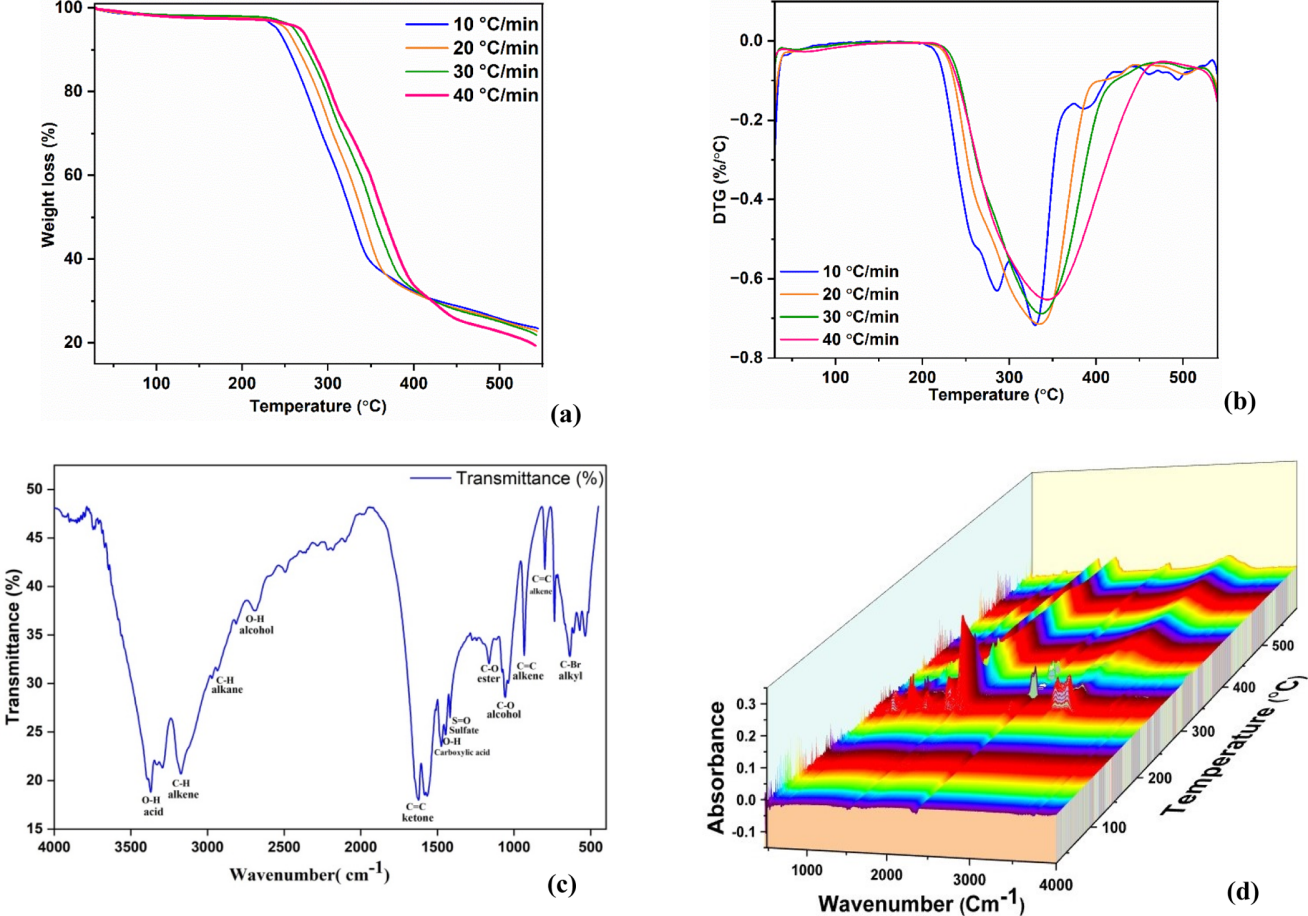
(a)­TG, (b) DTA, (c) FTIR (d) 3D FTIR diagram curves for Metformin.

All heating rates of the samples resulted in a similar DTG curve for the metformin pharmaceutical powder samples. As the heating rate increased, the curve began to move slightly in the direction of the greater temperature. This resulted from an increase in heat input as well as improved mass transfer.^45^ Figure 2b depicts the thermal degradation behavior of the metformin sample’s DTG curve. There was just one major peak in each curve, which was the result of the primary breakdown phase of the pharmaceutical product. The major peak in each curve indicates the degradation that relates to the higher rate of percentage mass loss. The major peak temperature for thermal decomposition of metformin samples at 10, 20, 30, and 40 °C/min was 330.4 °C, 334.7 °C, 337.1 °C, and 341.7 °C, respectively. This increase in temperature is brought on by the expansion of heat transfer that results from increased heating rates.^46^ The results are almost identical to another recently investigated pyrolysis of various material characteristics.^45,47,48^

### 4.2 FTIR Analysis of Metformin

A potent analytical method extensively utilized for the characterization of diverse materials is Fourier Transform Infrared (FTIR) spectroscopy. By measuring the interaction of infrared radiation with a sample, the various kinds of molecular bonds and functional groups that are present in the substance can be determined with great accuracy by FTIR spectroscopy. The intensity of light at different wavelengths is recorded, resulting in an FTIR spectrum. The place of position, intensity, and shape of the absorption peaks in the FTIR provide insights into the functional groups and chemical bonds within the sample.^46^

The obtained FTIR spectrum of Metformin is shown in Figure 2c,d. It exhibited distinct absorption peaks at specific wavenumbers. We can determine the presence of several functional groups in the metformin sample by examining these peaks. An O–H stretching vibration causes the peak to be visible at 3293.6 cm^–1^, which denotes the presence of hydroxyl groups like phenolic or alcoholic compounds.^45^ In addition, the peaks at 3173.2 cm^–1^ are attributable to strong C–H stretching vibrations, which suggest that Metformin may include alkenes. Alkanes may be present as evidenced by a vibration at 2934.9 cm^–1^, which is consistent with strong C–H stretching vibrations. Furthermore, the peak at 2687.2 cm^–1^ represents a stretching vibration of O–H bonds, implying the presence of alcoholic functional groups.^49^ The peak at 1620.7 cm^–1^ corresponds to a strong C=C stretching vibration, demonstrating the presence of esters or ketones. The peak at 1620.7 cm^–1^ corresponds to a C=C stretching vibration, indicating the presence of ketones, or esters.^50^ The peak at 1471.7 cm^–1^ corresponds to an O–H stretching vibration, indicating the presence of carboxylic acid groups.^50^ Furthermore, the peaks at 1163.6 cm^–1^ and 1064.6 cm^–1^ represent a stretching vibration of C–O bonds, implying the presence of ester or alcoholic functional groups in the Metformin. The peaks at 933.7 cm^–1^ and 799.8 cm^–1^ represent a strong stretching vibration of C=C bonds, implying the presence of alkenes in the Metformin.^51^ In summary, the FTIR analysis of metformin powder reveals the presence of hydroxyl groups (phenolic or alcoholic), carbonyl groups (aldehydes, ketones, or esters), and ether/alcohol groups. These functional groups contribute to the overall chemical composition of metformin powder and may have implications for its potential applications in various industries. These FTIR results are in good agreement with the reported literature.^48^

Figure 2a–d TGA, DTA, and FTIR curves of metformin illustrate its thermal decomposition characteristics under varying heating rates. These results directly support one of the objectives, “the thermal decomposition behavior of metformin pharmaceutical waste at different heating rates using TGA and DTA techniques,” helping to understand the material’s stability and thermal degradation profile.

### 4.3 Kinetic Parameters Analysis

To achieve an effective industrial application, one needs to know the kinetics of the pyrolysis process. Designing reactors where thermal conversions take place requires this approach. TGA curves produced at different levels of heating were used to calculate the temperature related to a constant value corresponding to the conversion percentage (α) for each heating rate.^48^ The characteristics of microscopic reactions that take place during the pyrolysis of materials can be determined through iso-conversional methods, as proposed by the ICTAC Kinetics Committee.^42^ The model-free iso-conversional methods (FRD, Starink, FWO, and KAS) were applied to obtain kinetic parameters. Most activation energy determinations exhibited high *R* ^2^ values, supporting the reliability of the kinetic data over a broad conversion range. However, slightly lower *R* ^2^ values were observed at low and high conversion, indicating reduced linearity that may arise from overlapping or secondary processes.

Molecular collision theory can render the obvious activation energy obtained using iso-conversional methods understandable even in the absence of any physical significance. Figure 3a–d show the KAS, FWO, Starink, and FRD linear plots for the pharmaceutical powder samples of Metformin, respectively. These plots were generated from the final equations reflecting the various linear regression-based approaches. The average frequency factors (A) for KAS, FWO, Starink, and FRD methods are 2.89 × 10^9^, 5.74 × 10^9^, 2.87 × 10^9^, and 1.77 × 10^11^ S^1–^, respectively. Due to the large range of values surrounding the critical threshold of 10^9^ S^1–^, the values of the frequency factor for the current investigation show that pyrolysis reactions are complex.^45^ Figure 3a–d present the linear plots from KAS, FWO, Starink, and FRD methods for the metformin samples. These are generated from isoconversional models and correspond to the objective “The kinetic parameters such as activation energy (*E*_a_), pre-exponential factor (A), and reaction model using model-free kinetic methods (KAS, FWO, Starink, and FRD), focusing on deriving kinetic parameters for pyrolysis”.

**Figure 3:**
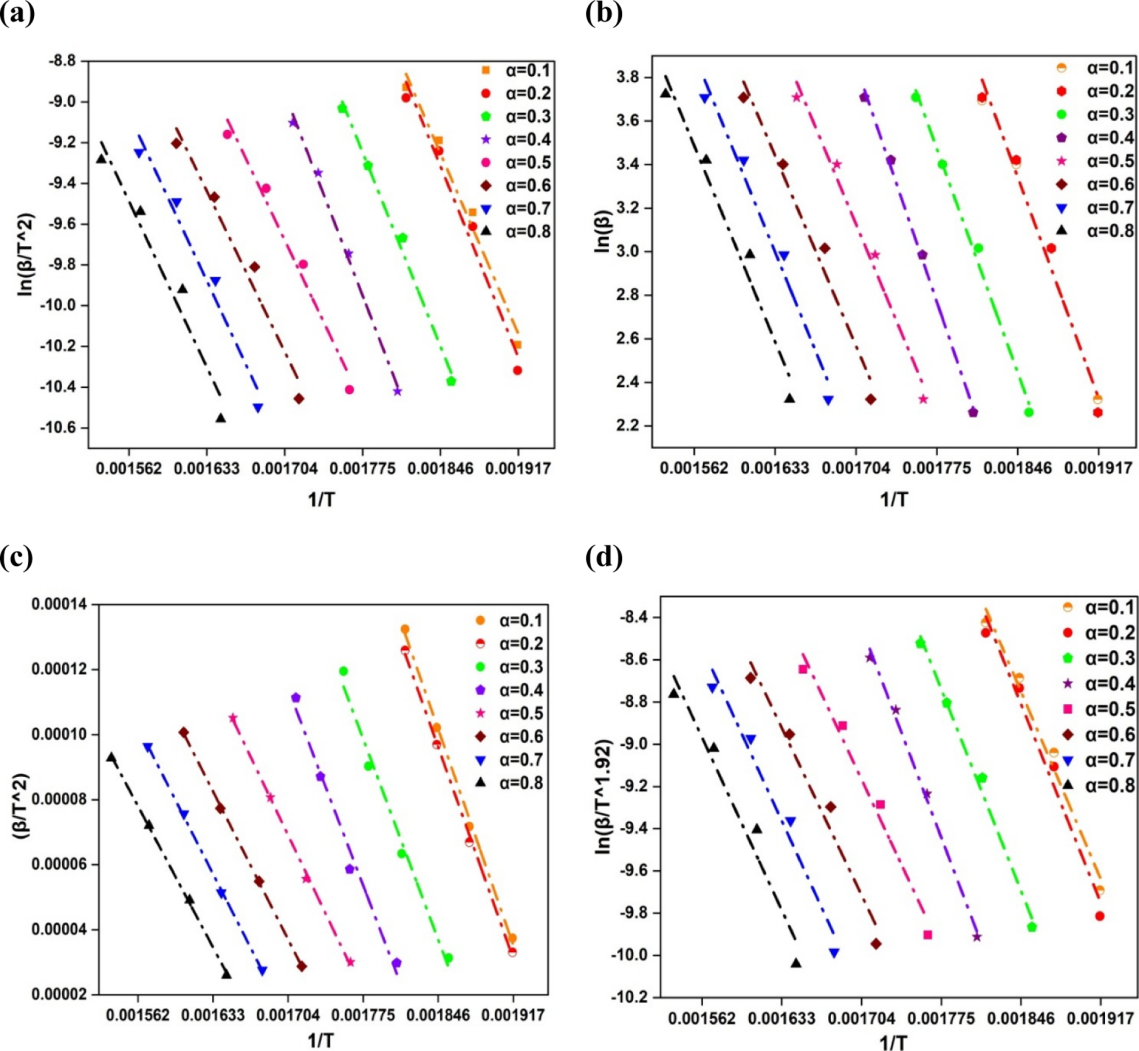
Isoconversional model plots (a) KAS, (b) FWO, (c) FRD, and (d) Starink.

The activation energy is the least amount of energy required to start the reaction. It serves as an estimate for the degree of thermal deterioration. The reaction is more likely to occur when the activation energy is lower.^52^ The average Activation energy values for metformin pharmaceutical powder samples pyrolysis from KAS, FWO, Starink, and FRD methods are 101.4, 105.8, 101.4, and 111.1 kJ/mol, respectively. As can be observed, there the variances in the average activation energy estimated using the KAS, FWO, Starink, and FRD techniques, because of various approximations used in the temperature integral calculation. This supports the ICTAC committee’s advice to use model-free techniques and the validity of the findings to their dependability.^42^ The range of Activation energy of KAS, FWO, Starink, and FRD methods is 92.8–126.8 kJ/mol, 99.2–20.0 kJ/mol, 92.8–116.7 kJ/mol, and 86.7–135.7 kJ/mol, respectively. To date, only a single study has investigated pharmaceutical pyrolysis using the KAS and FWO methods, reporting activation energies of 125.9 and 128.8 kJ/mol, respectively, for paracetamol.^48^ These values are notably higher than those observed in our current study on metformin, where the activation energy values ranged from 86.7 to 135.7 kJ/mol across four iso-conversional models (KAS, FWO, Starink, and FRD). The relatively lower average activation energies (101.4–111.1 kJ/mol) in our study indicate that metformin may decompose more readily than paracetamol under similar pyrolytic conditions, possibly due to differences in molecular structure and thermal stability. This wide range in activation energy values may be caused by the need for several reaction steps due to the various degradation paths.^53^ As shown in Figure 4a, Activation energy vs Conversion curves for all the models, the activation energy varied by ± 5 kJ/mol. All activation energy values had strong *R* ^2^ values (>0.97), demonstrating the reliability of the acquired results. Molecular collision theory is capable of rendering the obvious activation energy obtained using iso-conversional methods understandable, even though the absence of any physical significance.^54^ According to the molecular collision theory, random pyrolysis only affected a small number of special molecules, increasing their kinetic energy and enabling them to start reactions that broke earlier chemical associations.^55^ The kinetic parameters agreed with the literature that estimated the kinetic variables of the thermal degradation of different pharmaceuticals in a nitrogen environment employing the KAS and FRD methods.^56^

**Figure 4:**
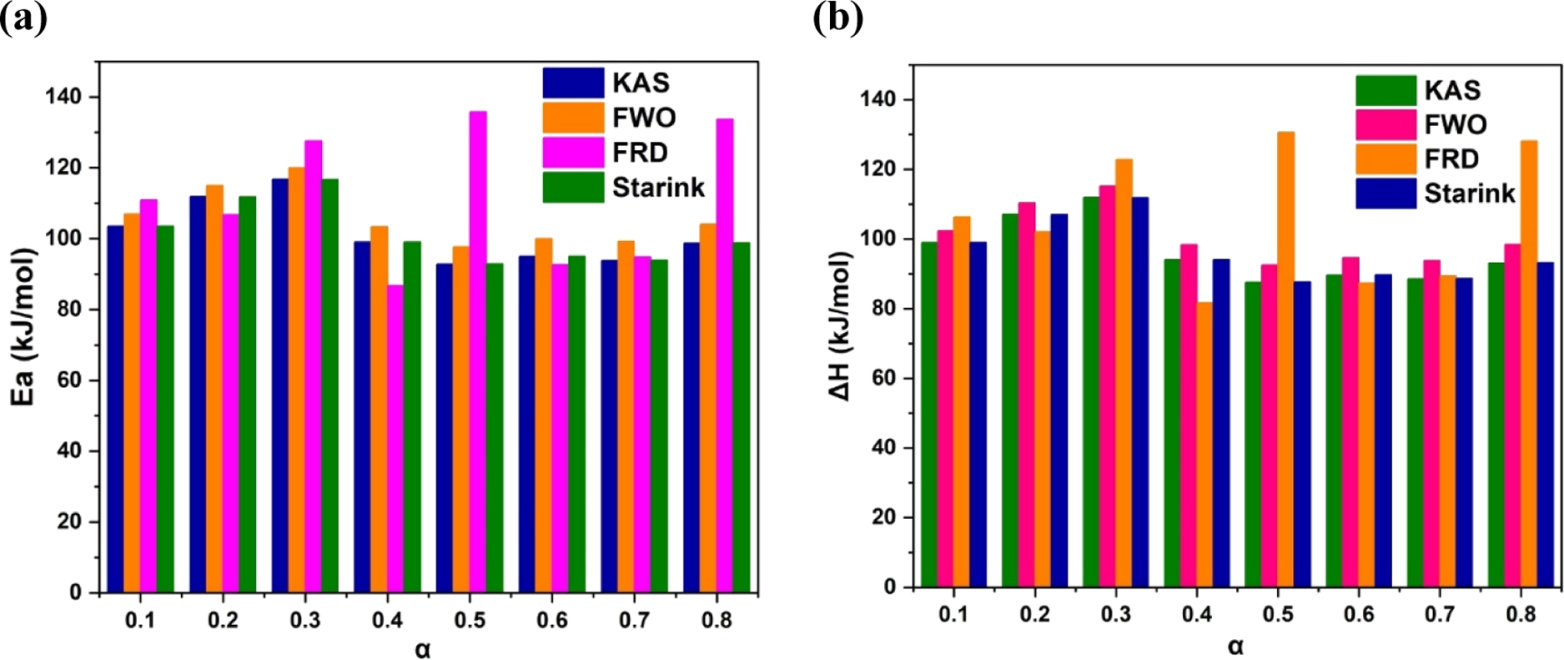
(a) Activation energy vs conversion curve, (b) enthalpy vs conversion curve for all the models.

The relationship between conversion (α) and temperature for all four heating rates was studied and shown in Figure 5. It is observed from Figure 5 that, for a particular conversion, the corresponding temperature increases systematically with the heating rate. This shift to higher temperatures at elevated heating rates is attributed to the reduced residence time available for heat transfer and reaction progression, resulting in thermal lag. Such trends are typical in thermogravimetric studies and support the kinetic analysis performed using the model-free methods. Figure 5 shows the conversion (α) versus temperature curves at different heating rates, offering a detailed insight into the decomposition behavior. This indirectly complements the objective by supporting the interpretation of product evolution and kinetic shifts under thermal treatment.

**Figure 5:**
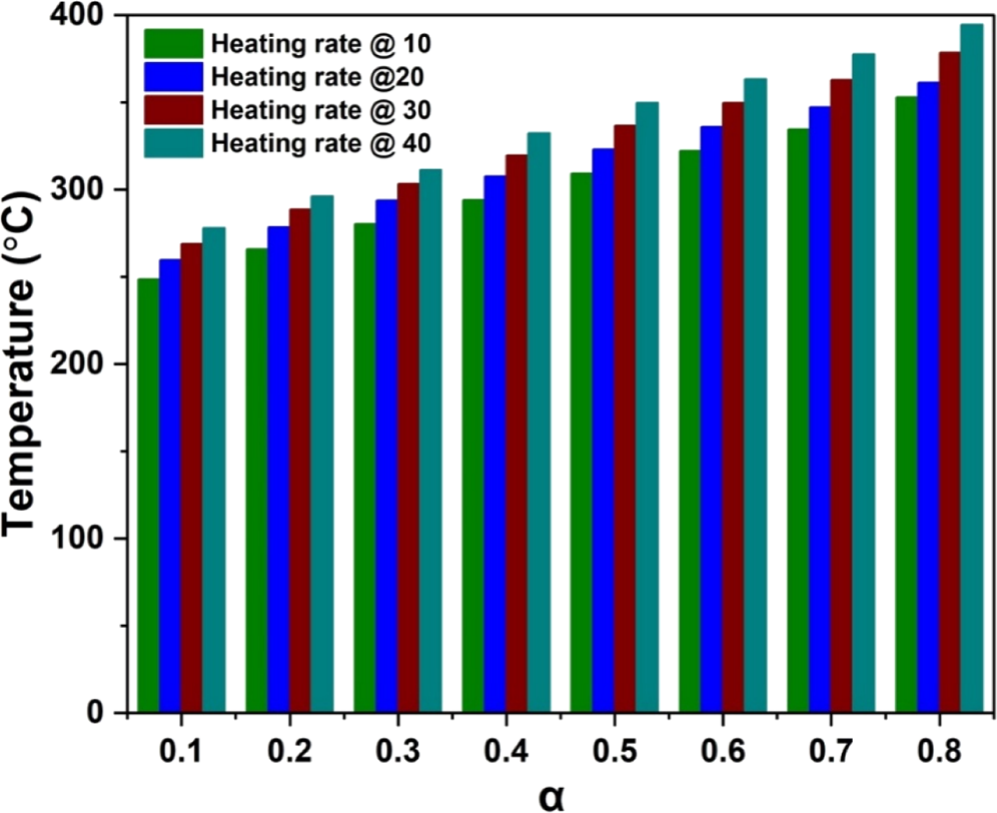
Conversion (α) versus temperature curves for all heating rates.

### 4.4 Thermodynamic Factors Analysis

Thermodynamic factors control the pyrolysis cycle’s efficiency and its evaluation of heat.^43^ Calculating the energy differential between the raw feedstock and the activated composite is simple to understand since the whole amount of heat produced during a reaction is accessible. Thermodynamic parameters for the metformin pharmaceutical powder sample’s thermal degradation were calculated. The thermodynamic factors Δ*G*, Δ*H*, and Δ*S* are determined by eqs 18, 19, and 20 are shown in Table 1. The average enthalpy (Δ*H*) for metformin pharmaceutical powder samples pyrolysis from KAS, FWO, Starink, and FRD methods is 96.3, 100.7, 96.4, and 106.0 kJ/mol, respectively. It suggests that only a little potential energy is needed for the reaction to take place.^57^ As shown in Figure 4b, Enthalpy vs Conversion curve for all the models, the change in enthalpy (Δ*H*) varied by ± 5 kJ/mol. The average enthalpy change across methods ranged from 96.3 to 106.0 kJ/mol, reflecting the endothermic nature of metformin pyrolysis. Compared to the values observed in the paracetamol study,^48^ the present study Δ*H* values suggest relatively lower energy requirements, reinforcing the idea of easier decomposition for metformin.

**Table 1:** KAS, FWO, FRD and Starink Methods for Kinetic and Thermodynamic Factors

model	α	*E*_a_ (kJ/mol)	*A* (min^–1^)	Δ*H* (kJ/mol)	Δ*G* (kJ/mol)	Δ*S* (J/(mol K))	R2
KAS	0.1	103.599	9.98 × 10^8^	99.018	152	–86.1	0.98
	0.2	111.845	5.55 × 10^9^	107.112	151	–72.08	0.99
	0.3	116.806	1.54 × 10^10^	111.947	151	–63.79	1
	0.4	99.061	3.96 × 10^8^	94.028	152	–94.53	0.97
	0.5	92.809	1.08 × 10^8^	87.63	152	–105.56	0.98
	0.6	94.962	1.69 × 10^8^	89.67	152	–102.02	0.97
	0.7	93.937	1.37 × 10^8^	88.527	152	–103.99	0.96
	0.8	98.724	3.74 × 10^8^	93.172	152	–95.84	0.91
	Avg.	101.468	2.89 × 10^09^	96.388	152	–90.49	0.97
FWO	0.1	106.95	1.99 × 10^9^	102.368	151	–80.35	0.98
	0.2	115.065	1.08 × 10^10^	110.331	151	–66.56	0.99
	0.3	120.013	2.98 × 10^10^	115.155	151	–58.31	1
	0.4	103.422	9.76 × 10^8^	98.389	151	–87.02	0.97
	0.5	97.739	3.01 × 10^8^	92.56	152	–97.05	0.98
	0.6	99.994	4.80 × 10^8^	94.702	152	–93.35	0.97
	0.7	99.231	4.10 × 10^8^	93.821	152	–94.86	0.96
	0.8	104.062	1.13 × 10^9^	98.51	151	–86.65	0.92
	Avg.	105.809	5.74 × 10^09^	100.73	151	–83.02	0.97
FRD	0.1	110.963	4.56 × 10^9^	106.382	151	–73.46	0.99
	0.2	106.753	1.94 × 10^9^	102.02	151	–80.81	0.99
	0.3	127.706	1.45 × 10^11^	122.848	151	–45.18	0.91
	0.4	86.736	3.05 × 10^7^	81.703	152	–115.84	0.97
	0.5	135.786	7.56 × 10^11^	130.607	150	–31.95	0.93
	0.6	92.668	1.05 × 10^8^	87.377	152	–105.99	0.93
	0.7	94.805	1.64 × 10^8^	89.395	152	–102.49	0.86
	0.8	133.758	5.07 × 10^11^	128.206	150	–35.89	0.64
	Avg.	111.147	1.77 × 10^11^	106.067	151	–73.95	0.89
Starink	0.1	103.572	9.92 × 10^8^	98.991	152	–86.15	0.98
	0.2	111.799	5.50 × 10^9^	107.066	151	–72.15	0.99
	0.3	116.751	1.53 × 10^10^	111.893	151	–63.88	1
	0.4	99.084	3.98 × 10^8^	94.051	152	–94.49	0.97
	0.5	92.866	1.09 × 10^8^	87.687	152	–105.46	0.98
	0.6	95.02	1.71 × 10^8^	89.728	152	–101.92	0.97
	0.7	94.007	1.39 × 10^8^	88.597	152	–103.87	0.96
	0.8	98.788	3.79 × 10^8^	93.237	152	–95.73	0.92
	Avg.	101.486	2.87 × 10^09^	96.406	152	–90.46	0.97

The Gibbs free energy represents the increase in the reaction system’s overall energy following the appearance of the reagents and the production of the activated complex.^58^ Average Gibbs free energy for metformin pharmaceutical powder samples, pyrolysis of KAS, FWO, Starink, and FRD methods are 152.0, 151.0, 152.0, and 151.0 kJ/mol, respectively. When Δ*G* is positive, it means that the reaction is not spontaneous and needs energy to take place. The Gibbs free energy values in our study averaged between 151.0 and 152.0 kJ/mol, which, although slightly lower than the values reported for paracetamol (∼166–172 kJ/mol), study.^48^ Still indicates nonspontaneous behavior under standard conditions. However, such Δ*G* values are consistent with thermally driven processes where external energy (heat) is required to initiate decomposition.

The degree of disorder in the system is indicated by entropy.^59^ Low activation entropy is a sign that the material has just experienced a physicochemical aging process that has brought it very close to its specific thermodynamic equilibrium. In this instance, it will take longer to create an active complex because of the low level of reactivity of the components. If high activation entropy is detected, the substance is out of thermodynamic equilibrium. In this way, the mechanism of reaction will react more quickly to produce the activated compound due to the high reactivity and consequent need for short reaction times.^58^ Therefore, as a change in entropy (Δ*S*) increases, the system becomes more reactive. The average change in entropy (Δ*S*) for metformin pharmaceutical powder samples pyrolysis from KAS, FWO, Starink, and FRD methods are −90.49, −83.02, −90.46, and −73.95 kJ/mol, respectively. These negative Δ*S* values imply a decrease in disorder during the transition from reactants to activated complexes, consistent with bond cleavage and the formation of more ordered intermediate species.^48^ In contrast, the paracetamol study also reported similar negative entropy changes, further validating our findings.

### 4.5 Pyrolysis Product Yields

The pyrolysis of metformin pharmaceutical powder was processed using a microwave-enhanced pyrolysis reactor at a fixed microwave power of 300 W and an operating temperature of 550 °C. To obtain pyrolysis product yields, 10 g of metformin pharmaceutical powder was blended with a fixed quantity of 10 g of the susceptor. For the pyrolysis process to produce the best product spectrum flexibility, one of the most crucial variables is the heating rate. For each pyrolysis experiment, the average heating rate was calculated using the measured operating temperature and the corresponding pyrolysis (residence) time. In metformin pharmaceutical powder pyrolysis, the calculated average heating rate was 59.1 °C/min, and it required 9 min to reach the operating pyrolysis temperature of 550 °C. The metformin pharmaceutical powder pyrolysis product masses and yields were calculated using eqs 1–6. In comparison to the previous study,^48^ which reported pyrolysis yields of 62.5 ± 0.80 wt % liquid, 31.7 ± 0.82 wt % gas, and 5.9 ± 0.58 wt % char, the present study on metformin pyrolysis demonstrated significantly different product distributions. Our results showed a bio-oil (liquid) yield of 46.6 wt %, a gas yield of 49.84 wt %, and a char yield of only 3.6 wt %. In terms of mass, the pyrolysis produced 4.66 g of bio-oil, 4.98 g of gas, and 0.36 g of char, indicating a higher proportion of volatile components in metformin pyrolysis compared to the reference material. These differences may be attributed to the structural and compositional variations between the pharmaceutical compounds studied. Table 2 shows the mass of each product and its corresponding yield. Additionally, the produced bio-oil was characterized by using GC–MS analysis.

**Table 2:** Three-phase Product Yields From Microwave Assisted Pyrolysis of Metformin

s.no	pyrolysis products	yields (wt %)	mass (g)
1	char	3.6 ± 0.54	0.36 ± 0.005
2	liquid	46.6 ± 0.84	4.66 ± 0.04
3	gas	49.8 ± 0.82	4.98 ± 0.02

### 4.6 Bio-Oil Yield Characterization by GC–MS

Gas chromatography with an MS detector for substance recognition was used to characterize the liquid pyrolysis products to further identify the chemical components that were formed. The chromatogram examined from the analysis of the diluted and purified liquid sample is shown in Figure 6. The liquid pyrolysis sample contained 21 major peaks with eluted masses that were higher. Table 3 enlisted all the compounds, their relative area of the peaks’ chemical names, and their chemical formulas. The chemicals identified by the GC–MS examination contain the functional groups identified by the TGA-FTIR study.

**Figure 6:**
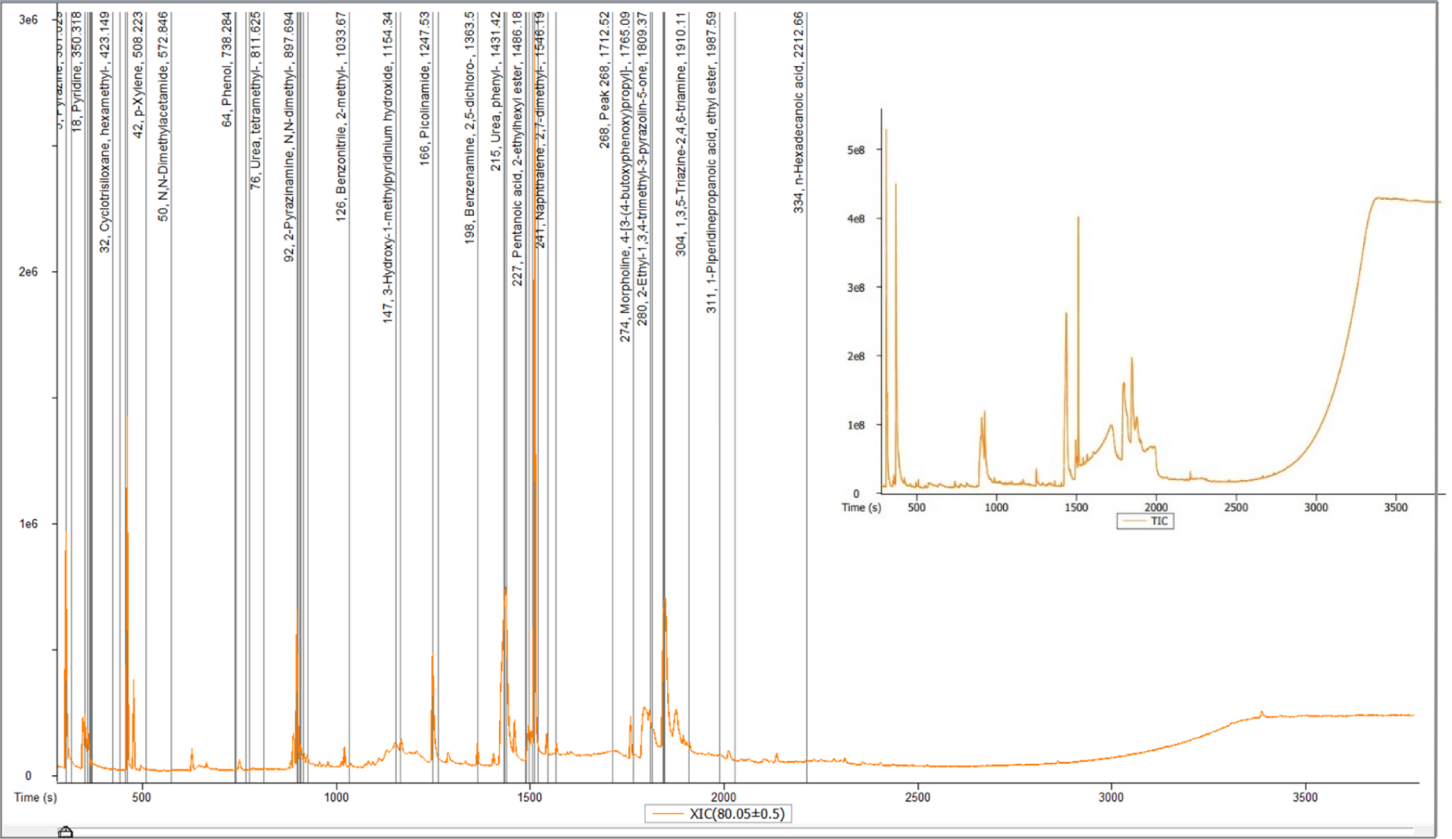
Chromatogram of metformin pyrolysis liquid.

**Table 3:** List of Compounds and Peak Area of GC–MS Analysis of Pyrolysis Liquid

s. no	residence time (s)	chemical name	molecular formula	area % of peak
1	307.9	acetonitrile, (dimethyl amino)	C_4_H_8_N_2_	14.12
2	315.2	4,7-dimethyl-5H,6H-2,1,3,4,7-^1,2,5^oxadiazolo^3,4–b^pyrazin-1-one	C_6_H_10_N_4_O_2_	2.10
3	368.1	2-amino-4-methyl pyrimidine	C_5_H_7_N_3_	5.147
4	368.7	2-pentanone, 4-methoxy-4-methyl-	C_7_H_14_O_2_	39.94
5	572.8	*N*,*N*-dimethylacetamide	C_4_H_9_NO	1.50
6	924.9	urea, trim ethyl-	C_4_H_10_N_2_O	3.081
7	1247.5	picolinamide	C_6_H_6_N_2_O	0.594
8	1434.9	2-(diacetoxymethyl)-5-nitrofuran	C_9_H_9_NO_7_	2.581
9	1846.0	oxalic acid, allyl pentyl ester	C_10_H_16_O_4_	1.589
10	1910.1	1,3,5-triazine-2,4,6-triamine	C_3_H_6_N_6_	1.158
11	2028.1	propanal, 2-methyl-	C_4_H_8_O	0.133
12	1809.3	2-ethyl-1,3,4-trimethyl-3-pyrazoline-5-one	C_8_H_14_N_2_O	0.257
13	1814.5	3-hepten-2-one, 3-propyl-	C_10_H_18_O	0.19
14	367.7	guanidine carbonate	C_3_H_12_N_6_O_3_	0.35
15	900.1	ethanone, 1-(1H-pyrrole-2-yl)-	C_6_H_7_NO	0.16
16	775.2	4-aminopyrimidine	C_4_H_5_N_3_	0.52
17	423.1	cyclotrisiloxane, hexamethyl-	C_6_H_18_O_3_Si_3_	0.45
18	1248.4	fluoxetine	C_17_H_18_F_3_NO	0.348
19	358.0	cyanamide, dimethyl-	C_3_H_6_N_2_	0.346
20	358.0	iron, tetra carbonyl(pyridine)-, (tb-5–12)-	C_9_H_5_FeNO_4_	0.24
21	350.3	pyridine	C_5_H_5_N	0.20

The *N*,*N*-Dimethylacetamide (Metformin) itself, as well as 4-methylamino-2-amino-1,3,5-triazine, methyl quinolone, 2-Pentanone, 4-methoxy-4-methyl-, and guanidine carbonate, may all be responsible for the GCMS peaks that correspond to area percentage of peaks. 4-Methylamino-2-amino-1,3,5-triazine is a direct product of the oxidation of Metformin.^60^ Superoxide radicals are weak metformin oxidation initiators under similar conditions, indicating that Metformin is not a potent antioxidant. The immediate byproducts of Metformin’s thermal breakdown include acetonitrile, (dimethyl amino), 2-Pentanone, 4-methoxy-4-methyl, and 2-(Diacetoxymethyl)-5-nitrofuran, demonstrating that at least some of the API was decomposed down during pyrolysis.^25^ Picolinamide, a structural isomer of Metformin, was the other metformin-derived substance found in the sample and whose peaks made up 0.59% of the overall chromatogram area. Picolinamide has been shown to have C–H activation and more generally to be involved in the production of heteroaryl amides.^61^ It has been shown that isomerization occurs during the pyrolysis of several different substances, including toluene, pyrazine, and benzene.^62^

The presence of propanoic, oxalic, and benzoic acids might also be indicators of acetaminophen breakdown. They were included because their existence was compatible with research that revealed that acetaminophen’s breakdown products comprised a variety of carboxylic acids.^26^ Furthermore, nitric acid can result from the thermal breakdown of glycerol triacetate, another excipient indicated on the utilized tablet.^63^ It is noticeable that 2-Pentanone, 4-methoxy-4-methyl-, accounts for a sizable portion (totaling 39.94% of the chromatogram’s total area). This might be because the solvent for various resin coatings, which is indicated as an excipient of the tablets under analysis, was included.^64^ Additionally, throughout the pyrolysis process, the organic carbonaceous molecules are polymerized. It may be responsible for the occurrence of these long-chain alkanes.^64^ Guanidine carbonate, which is closely related to urea and may be a sign of the thermal breakdown of cyanamide, is also present in the sample.^65^ Furthermore, it should be noticed that the peak area percentage that corresponds to Fluoxetine makes up 0.35% of the overall area of the chromatogram, indicating that the API makes up a smaller portion of the pyrolysis liquid. The primary indication for fluoxetine is depressive illness.^66^

It is therefore possible that the pyrolysis of unneeded or expired pharmaceuticals might be employed as a procedure that results in the reuse or recycling of the API for various medicinal products after it has been isolated from the other components of the matrix. However, as throwing Metformin in the environment has been linked to several negative effects, including the toxicity of wastewater to the environment, it would be essential to remove any residues of the drug from the API that is to be recycled.

### 4.7 Artificial Neural Network Modeling

An ANN is a deep linear information processing network that simulates the nervous system and brain by modeling the behavior of neurons using perceptron technology.^67^ To get the desired outcome, ANN models develop patterns based on past data. This algorithm was used to decrease the discrepancy between experimental and predicted results by modifying the weight coefficient of each neuron.^68^ ANN analysis is predicted to be employed to determine the autotuning of the pyrolysis reactor’s optimum parameters.^69^ To better understand the pyrolysis of Metformin, a modeling of the experimental data applying an ANN model was used to predict outcomes using machine learning. An artificial neural network was developed to predict weight loss using experimental data from TG analysis. The input variables included temperature (°C) and heating rate (°C/min), while the output was fractional mass loss. A total of 840 data points were used, split into 60% for training, 20% for testing, and 20% for validation. A feed-forward back-propagation network with one hidden layer comprising 4 neurons was employed, and the network was trained using the Bayesian regularization algorithm (trainer) in MATLAB. The optimal number of neurons was selected based on minimizing mean squared error (MSE) through trial and error. The ANN model demonstrated good predictive capability on the test data set.

Since there is no accurate approach for finding how many neurons are present in hidden layers, a trial-and-error approach was employed. Figure 7 illustrates the performance curve, error histogram, and analysis of training, validation, and testing. Figure 7a,b demonstrated that there was little variance between the objective and output values, and the subsequent histogram of error distribution showed. The ANN model for predicting weight loss during metformin pyrolysis exhibited excellent performance, as indicated by the regression plots (Figure 7a), error histogram (Figure 7b), and learning curve (Figure 7c). The high correlation coefficients (*R* ≈ 0.9998 for training, validation, and testing) suggest a near-perfect agreement between predicted and actual values, demonstrating the model’s ability to capture the nonlinear thermal degradation behavior accurately. The error histogram shows a normal distribution of errors centered on zero, with minimal deviations, further reinforcing the model’s precision. Additionally, the mean squared error (MSE) plot indicates that the model reached its optimal performance at epoch 13, where the validation error stabilized at 3.2368 × 10^–5^, confirming effective training without overfitting. These results highlight the ANN’s capability as a reliable predictive tool for pyrolysis kinetics, reducing the need for extensive experimental trials while improving process efficiency in pharmaceutical waste management. The model’s best performance at the lowest value of MSE was obtained after an adequate number of repetitions, as shown in Figure 7c. The findings are quite positive, as models provide a better understanding of the pyrolysis of biomass sources and are almost perfectly in line with some latest studies.^45^ Developing an empirical connection between the inputs and the output employing the developed ANN model is beneficial.

**Figure 7:**
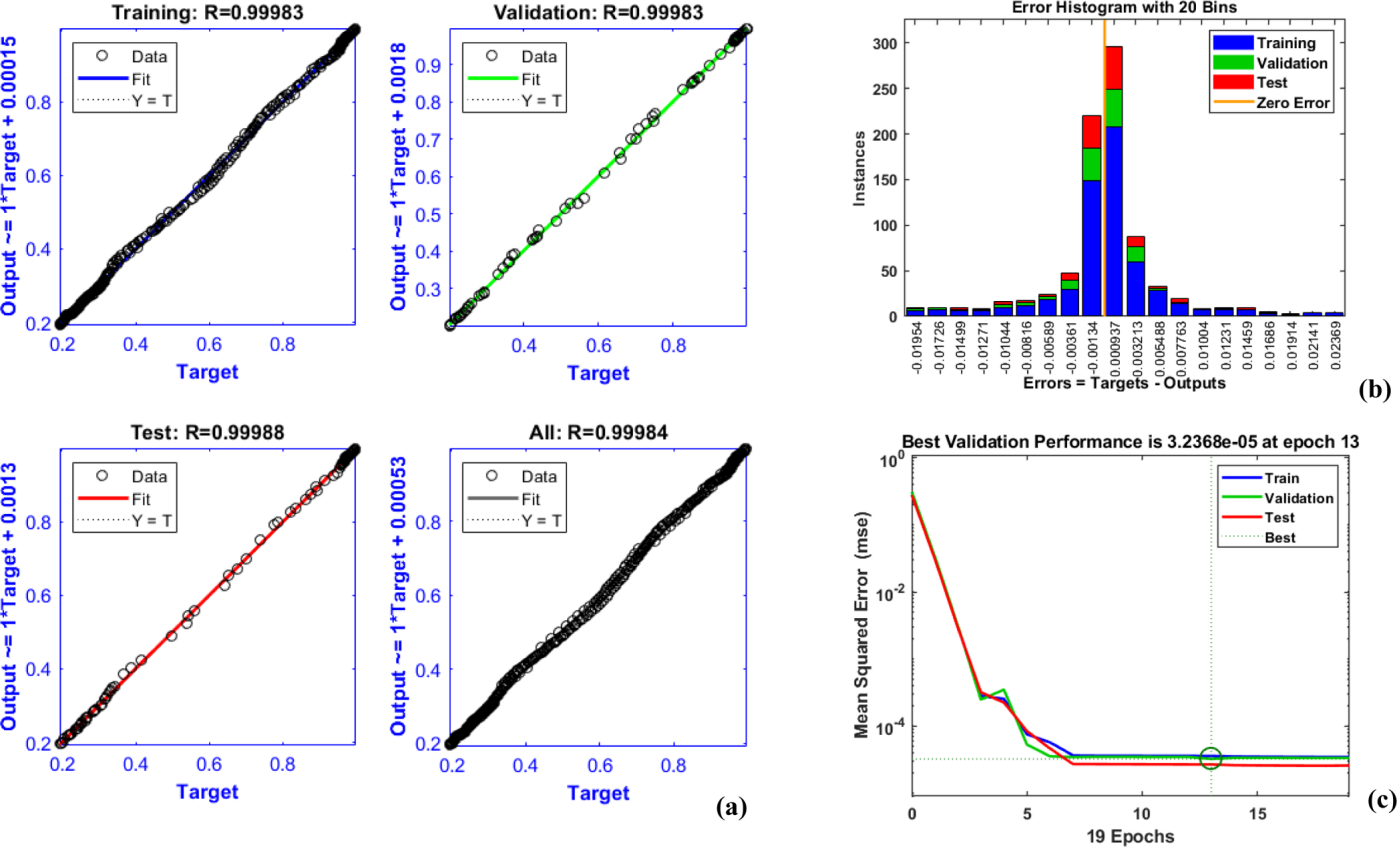
(a) Analysis of training, validation, and testing, (b) error histogram (c) performance curve.

## 5 Conclusions

The purpose of this study was to investigate pyrolysis as an alternative way of treating pharmaceutical wastes designed to promote the development of a circular economy. The most popular diabetic medication, Metformin, was subjected to a pyrolysis experiment at 550 °C. At four different heating rates, the pyrolysis of the Metformin was studied using TGA to examine its thermal breakdown behavior. The major peak temperature for thermal decomposition of metformin samples at 10, 20, 30, and 40 °C/min was 330.4 °C, 334.7 °C, 337.1 °C, and 341.7 °C, respectively. To determine the Δ*G*, Δ*H*, and Δ*S* as well as kinetics, four isoconversional methods such as KAS, Flynn-Wall-Ozawa FWO, Tang, Starink, and Boswell, were used. Activation energy values for metformin pharmaceutical powder samples pyrolysis from KAS, FWO, Starink, and FRD methods are 101.4, 105.8, 101.4, and 111.1 kJ/mol, respectively. Average Gibbs free energy (Δ*G*) for metformin pharmaceutical powder samples pyrolysis of KAS, FWO, Starink, and FRD methods are 152.0, 151.0, 152.0, and 151.0 kJ/mol, respectively. According to the study of the pyrolysis liquid yield by GC–MS, the API made up the majority of the total area of the chromatogram peaks while only a minor percentage corresponded to long-chain alkanes with fuel-like properties. The use of ANN also showed a strong association with the results of the experiments, enhancing the validity of the analysis.

Future research may focus on scaling up the pyrolysis process for pharmaceutical waste and integrating it with continuous reactor systems for industrial feasibility. Additionally, the application of advanced hybrid machine learning models could further improve kinetic predictions and process optimization. Investigating the environmental impact and potential reuse of pyrolysis products in pharmaceutical or chemical industries could also open up new avenues aligned with circular economy principles.

## Nomenclature

*E*_a_: activation energy of the reaction (kJ/mol)
*f*(α): reaction model function, dependent on fractional conversion (α)
K+: metal ions
*K*_b_: Boltzmann constant
*k* (*T*): reaction rate constant at temperature T
KOH: potassium hydroxide
OH^–^: hydroxide ions
*R*: universal gas constant (8.314 J/mol.K)
*t*: time (s)
*T*: temperature (K or °C)
*T*_m_: peak temperature from DTG curve (K or °C)
&alpha: fractional conversion
*w*_*t*_: sample mass at time *t*
*w*_0_: initial sample mass
*w*_f_: final sample mass
*h*: Planck’s constant
AI: artificial intelligence
ANN: artificial neural network
API: active pharmaceutical ingredient
DCM: dichloromethane
DOE: design of experiments
DTG: derivative thermogravimetric
EPA: Environmental Protection Agency
FTIR: Fourier transform infrared spectroscopy
FWO: Flynn–Wall–Ozawa method
GC–MS: gas chromatography–mass spectrometry
HHV: higher heating value
KAS: Kissinger–Akahira–Sunose method
MAP: microwave-assisted pyrolysis
ML: machine learning
MLR: multilinear regression
RF: random forest
RSM: response surface methodology
TGA: thermogravimetric analyzer
TG–DTA: thermogravimetric–differential thermal analysis
FRD: Friedman method
FWO: Flynn–Wall–Ozawa method
Starink: Starink method
KAS: Kissinger–Akahira–Sunose method
*A*: pre-exponential factor (s^–1^).
